# P-484. Global Economic Burden Of Pneumococcal Disease In Children — Lessons Learned From Economic Evaluations Of Pneumococcal Vaccines

**DOI:** 10.1093/ofid/ofaf695.699

**Published:** 2026-01-11

**Authors:** Min Huang, Jipan Xie, Walter A Orenstein, Hela Romdhani, Yan Song, Elamin Elbasha, Matthew S Kelly

**Affiliations:** Merck & Co., Inc., Rahway, New Jersey; XL Source, Inc., Los Angeles, California; Emory University, Atlanta, Georgia; Analysis Group, Inc., Boston, Massachusetts; Analysis Group, Inc., Boston, Massachusetts, USA, Boston, MA; Merck & Co., Inc., Rahway, New Jersey; University of Arkansas for Medical Sciences, Little Rock, AR

## Abstract

**Background:**

Pneumococcal conjugate vaccines (PCVs) have significantly reduced the burden of pneumococcal disease worldwide. Recently, higher-valency PCVs have been approved and evaluated in economic models. This study summarizes the reported economic burden of pneumococcal disease in children in these studies.

**Methods:**

A targeted literature review was conducted using MEDLINE to identify cost-effectiveness analyses and cost modeling studies for pneumococcal disease (01/2021-06/2024). Fourteen studies conducted in 18 countries were identified. Cost inputs were extracted for invasive pneumococcal disease (IPD), inpatient pneumonia (IP), outpatient pneumonia (OP), acute otitis media (AOM), and post-meningitis sequelae (PMS, *ie*, disability and deafness). All costs were adjusted to 2023 US dollars using local consumer price indices and currency conversion rates. Median direct medical and indirect/non-medical costs and interquartile ranges (IQR) were reported separately for the US and other countries (including Canada and countries in Asia, Europe and Oceania).

**Results:**

Direct costs per episode in the US were $56,222 ($45,343-$56,222) for IPD, $20,691 ($20,011-$20,691) for IP, $681 ($578-$696) for OP, and $310 ($233-$431) for AOM; corresponding costs in other countries were $9,246 ($5,208-$16,867), $5,203 ($3,845-$6,510), $161 ($116-$293), and $223 ($106-$569). Lifetime direct costs in the US were $709,365 ($709,365-$749,027) for disability and $137,479 ($137,479-$145,165) for deafness, compared to $39,290 ($29,410-$679,937) and $101,249 ($54,245-$133,617) in other countries. Indirect/non-medical costs showed less variability, with costs of $1,544 ($881-$2,023) for IPD and IP, and $238 ($188-$438) for OP and AOM across all countries. Limited data suggested substantial indirect costs for PMS (range: $373,431-$1,331,166).

**Conclusion:**

Pneumococcal disease and PMS impose substantial economic burdens on the healthcare system and society, with the highest and lowest costs generally observed in the US and Japan, respectively. Data on contemporary costs in some countries and on the indirect costs of PMS are needed to more accurately quantify costs of these conditions and provide robust inputs for future economic evaluations.

**Disclosures:**

Min Huang, PhD, Merck & Co., Inc.: Min Huang is a full-time employee of Merck & Co., Inc.|Merck & Co., Inc.: Stocks/Bonds (Public Company) Jipan Xie, MD, PhD, XL Source, Inc.: I am an employee of XL Source, Inc., a consulting company that has provided paid consulting services for the study Walter A. Orenstein, MD, DSc (Hon), CureVac: Advisor/Consultant|Dynavax: Advisor/Consultant|Merck & Co., Inc.: Advisor/Consultant|Moderna: Advisor/Consultant|Sanofi: Advisor/Consultant Hela Romdhani, PhD, Analysis Group, Inc.: I am an employee of Analysis Group, Inc., a consulting company hat has provided paid consulting services to Merck & Co., Inc. Yan Song, PhD, Analysis Group, Inc.: I am an employee of Analysis Group, Inc., a consulting company hat has provided paid consulting services to Merck & Co., Inc. Elamin Elbasha, Ph.D., Merck & Co., Inc.: Employee|Merck & Co., Inc.: Stocks/Bonds (Public Company) Matthew S. Kelly, MD, MPH, Merck & Co., Inc.: Advisor/Consultant

